# The *DAX1* mutation in a patient with hypogonadotropic hypogonadism and adrenal hypoplasia congenita causes functional disruption of induction of spermatogenesis

**DOI:** 10.1007/s10815-012-9778-y

**Published:** 2012-05-05

**Authors:** Donata Ponikwicka-Tyszko, Malgorzata Kotula-Balak, Katarzyna Jarzabek, Barbara Bilinska, Slawomir Wolczynski

**Affiliations:** 1 Department of Biology and Pathology of Human Reproduction, Bialystok, Institute of Animal Reproduction and Food Research of Polish Academy of Sciences, Tuwima 10, 10-748 Olsztyn, Poland; 2 Department of Endocrinology, Institute of Zoology, Jagiellonian University, Gronostajowa 9, 30-387 Cracow, Poland; 3 Department of Reproduction and Gynaecological Endocrinology, Medical University of Bialystok, Sklodowskiej 24a, 15-276 Bialystok, Poland

## Introduction

Hypogonadotropic hypogonadism (HH) associated with adrenal hypoplasia congenita (AHC) is a very rare syndrome caused by mutation of *DAX1* (dosage-sensitive sex reversal-adrenal hypoplasia congenita critical region on the X chromosome) [19]. The *DAX1* is located on the chromosome X (Xp21.3–21.2) and contains two exons. It encodes a 470-amino acid protein which belongs to the nuclear hormone receptor superfamily (called DAX1). DAX1 is expressed in the adrenal cortex, pituitary and hypothalamus, gonadal cells such as Leydig and Sertoli cells, theca and granulosa cells and in germ cells [15, 26].

Patients with HH due to *DAX1* mutation exhibited azoospermia [4, 5, 17, 24]. Results of spermatogenesis induction using exogenous gonadotropin are unsatisfactory [4, 17, 24]. However, Frapsauce et al. [5] has recently presented the first birth after successful assisted reproductive technique (ART) using TESE-ICSI in a man with HH and AHC linked to a *DAX1* mutation. In this case, long period of gonadotropin treatment allowed for development of few mature spermatozoa obtained from testicular biopsy and used for ICSI [5].

Here, we report clinical and immunohistochemical studies of patient with HH and AHC due to deletion of the second exon of the *DAX1* in order to induce spermatogenesis by gonadotropins treatment.

## Materials and methods

### Direct sequencing of *DAX1* gene

Genomic DNA was extracted from peripheral blood leukocytes using the salting-out method. Both exons of *DAX1* gene were amplified by PCR using primers and conditions described previously [22]. Direct sequencing of PCR products was performed using the BigDye™ Terminator Cycle Sequencing Ready Reaction Kit 3.1 (Applied Biosystems). Both primers were used for sequencing both strands. The sequencing products were run on an ABI PRISM 3130 capillary automated sequencer (Applied Biosystems).

### Immunohistochemistry

For immunohistochemical studies 15 representative sections from the resected specimen were selected for each antigen studied. Following biological markers were investigated: gonadotropin receptors (LHR and FSHR), aromatase, estrogen receptors alpha and beta (ERα and ERβ), androgen receptors (AR), and gap junction protein connexin 43 (Cx43). The whole immunohistochemical procedure had been described in detail previously [2, 8, 16]. In brief, to achieve antigen retrieval slices were immersed in 10 mM citrate buffer (pH 6.0) and heated for 2 × 5 min in the microwave oven (750 W). Endogenous peroxidase activity was blocked with 3 % H_2_O_2_ in methanol for 15 min and nonspecific binding sites were blocked with 5 % nonimmune goat serum (v/v) for 30 min at room temperature. Thereafter, testicular sections were incubated overnight at 4°C in a humidified chamber in the presence with the following antibodies: rabbit polyclonal antibody against LHR (1:100; BIOTREND Chemikalien GmbH, Köln, Germany); goat polyclonal antibody against FSHR (N-20; 1:100; Santa Cruz Biotechnology, Inc., Santa Cruz, CA, USA); rabbit polyclonal antibodies against AR(N-20; 1:1000; Santa Cruz Biotechnology) and against Cx43 (1:2000; Sigma-Aldrich, St Louis, MO, USA) as well as mouse anti human monoclonal antibodies against P450 aromatase (1:100) and ERβ (1:50; Serotec, Düsseldorf, Germany) and against ERα (1:100; Dako, Glostrup, Denmark). Next, biotinylated secondary antibodies, horse anti-mouse IgG, horse anti-goat IgG or goat anti-rabbit IgG for monoclonal or polyclonal antibodies, respectively (1:400; Vector Labs., Burlingame, CA, USA) were applied. Finally, avidin-biotinylated horseradish peroxidase complex (Vectastain ABC Kit; 1:100; Vector Labs.) was used. After each step in these procedures, sections were carefully rinsed with Tris-buffered saline (TBS; 0.05 M Tris–HCl plus 0.15 M NaCl, pH 7.6). Bound antibody was visualized with TBS containing 0.05 % 3,3′-diaminobenzidine and 0.07 % imidazole for 3–4 min. Sections were counterstained with Mayer’s hematoxylin. Thereafter, sections were dehydrated, cleared in xylene, and mounted using DPX mounting medium (Fluka Biochemica, Steinheim, Germany). The slides were processed immunohistochemically at the same time with the same treatment so the staining intensity among different sections of the testis could be compared. Control sections included omission of the primary antibody and substitution by an irrelevant IgG. The sections were examined with a Leica DMR microscope (Leica, Microsystems GmbH Wetzlar, Wetzlar, Germany) using a Nomarski interference contrast. Experiments were repeated three times and the expression of each marker was assessed on the basis of visual examination of cytoplasmic or nuclear localization in 10 different fields on serial sections.

## Results

### Patient

At the age of 32 years the male patient consulted due to hypogonadotropic hypogonadism and infertility problems. At the age of 8 years he was diagnosed with adrenal hypoplasia congenita. Then substitution doses of 20 mg of hydrocortisone treatment every day was applied. Moreover, testosterone replacement therapy at a dose of 250 mg every three weeks since 16 age was administered. The patient is the only case with AHC and HH in his family. During physical examination and medical history both testes 2 cm^3^ in dimension, penis 4 cm in length, eunuchoid body proportions, height of 190 cm and hyperpigmentation of skin were observed. Azoospermia was revealed in routine semen analysis. Hormonal analysis showed: low LH level (0,1 IU/L, N:1.5–9.3 IU/L), low FSH level (0.4 IU/L, N: 1.4–18.1 IU/L) and low testosterone level (0.2 ng/mL, N: 2.4–8.2 ng/mL). A GnRH test was performed. Plasma LH concentrations did not increase after administration of pharmacological doses of GnRH, while FSH concentrations exhibited an increase to 1,3 mIU/mL in 60th minutes.

Previously in another clinic, in order to induce spermatogenesis, patient was treated for 4 months with 1500 IU hCG once a week, what we qualified as pretreatment. Then the standard our clinic treatment for each patient diagnosed with hypogonadotropic hypogonadism was applied. Patient was stimulated as follows: for 5 days, human menopausal gonadotropin (HMG, Menopur, Ferring) was administered at a dose of 75 IU FSH, and on the sixth day, hCG (Choragon, Ferring) was given at a dose of 1500 IU. In subsequent months, testosterone level was as follows: 3.63, 4.53, 4.23, 4.34 ng/mL (N: 2,2–9,8 ng/mL), whereas estradiol level was as follows: 56, 65, 75, 82 pg/mL (N: 10–52 pg/mL). According to our experience, after 4 months of such therapy in ejaculate should appear single spermatozoa, but semen analysis again showed azoospermia. Open testicular biopsy in both testes was performed, after obtain informed consent. Five samples from each testes were received. Complete lack of spermatozoa was confirmed by examination in light inverted microscope. Histological analysis was carried out after haematoxylin-eosin (H + E) staining and showed rare spermatogonia and spermatogenesis arrest. Testicular tissue was also fixed in 10 % (v/v) buffered formaldehyde solution for 48 h and then embedded in paraffin blocks at 56°C according to standard procedures for further immunohistochemical examination. All studies have been approved by the local Ethics Committee.

### *DAX1* gene mutation analysis

Genetic analysis, after obtain informed consent was conducted. Direct sequencing of *DAX1* allowed to exclude the presence of a point mutation in exon 1 *DAX1*gene. Deletion of exon 2 was confirmed by PCR reaction using two different sets of primers.

### Immunohistochemical studies

**Fig. 1:**
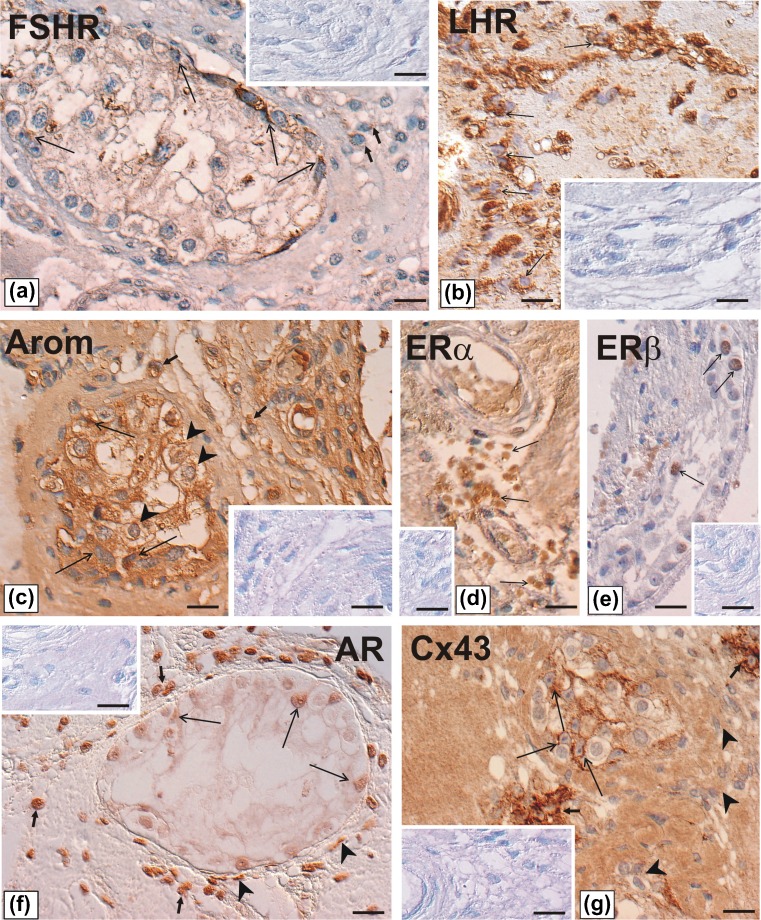
a-g. Immunohistochemical localization of FSHR, LHR, aromatase, ERα ERβ, AR, and Cx43 in well differentiated human Sertoli-Leydig cell tumor. Immunostainings were performed using monoclonal or polyclonal antibodies (for detail see the text) followed by anti-mouse, anti-goat or anti-rabbit IgG, and ABC/HRP visualized by DAB. Counterstaining with Mayer’s haematoxylin. Nomarski interference contrast. Bars = 20 μm. **a** Weak to moderate staining for FSHR in Sertoli cell cytoplasm (*long arrows*) and no staining in Leydig cells (*arrows*) are visible. **b** Strong staining for LHR in Leydig cell cytoplasm (*arrows*) is visible. **c** Cytoplasmic pattern of the staining for aromatase in Leydig cells (*arrows*) and Sertoli cells (*long arrows*) is visible. Note a few remained germ cells also positively stained (*arrowheads*). **d** Strong to very strong staining for ERα in nuclei of Leydig cells is visible. No staining is seen in the other cells. **e** Moderate to strong staining for ERβ in a few remained germ cells is visible. **f** All somatic cells are positively stained for AR. Note, weak staining in nuclei of Sertoli cells (*long arrows*), moderate to strong in Leydig and peritubular-myoid cells. **g** Strong to very strong signal for Cx43 between Sertoli and germ cells remained in the tubules (*long arrows*), and between neighboring normal-looking Leydig cells is visible (*arrows*). Note a diffuse pattern of the staining in hyperplastic Leydig cells (*arrowheads*). The expression of all the antigens was undetectable when the primary antibodies were omitted (see inserts in a-g)

Positive immunohistochemical staining for each antigen was found in all testicular sections with Leydig cell hypertrophy (Fig. 1a-g). Immunohistochemical analysis of the sections revealed differential expression of gonadotropin receptors, FSHR and LHR (Fig. 1a-b). Weak to moderate immunoexpression of FSHR was detected in the cytoplasm of some Sertoli cells, whereas Leydig cells were immunonegative (Fig. 1a). In contrast, in the sections stained for LHR, the Leydig cell cytoplasm was strongly immunopositive (Fig. 1b). Similar cytoplasmic pattern of the staining was found in both Leydig and Sertoli cells that displayed moderate to strong immunoreactivity for aromatase (Fig. 1c). A few remained germ cells were also immunopositive for aromatase. A strong to very strong immunoexpression, nuclear in pattern, was detected in all Leydig cells stained for ERα (Fig. 1d), whereas moderate to strong immunoexpression of ERβ was found in a few remained germ cells (Fig. 1e). Interestingly, all somatic cells: Sertoli, Leydig and peritubular-myoid cells expressed weak, moderate and strong staining for AR, respectively (Fig. 1f). Immunoreactive protein Cx43, as a strong signal, frequently linear in pattern, was present between Sertoli and germ cells remained in the tubules. Moreover, a very strong signal for Cx43 was also noted between neighboring normal-looking Leydig cells, whereas in numerous hyperplastic Leydig cells, the signal was of diffuse pattern (Fig. 1g). The expression of all the antigens was undetectable when the primary antibodies were omitted (see, inserts).

## Discussion

In the present study, molecular analysis revealed deletion of exon 2 of *DAX1* gene in patient with adrenal insufficiency and hypogonadotropic hypogonadisms. It has been shown that DAX1 is a transcriptional repressor of SF-1, ER, AR, PR, LHR1 [1, 7, 29], and repressor domain of DAX1 is located at its carboxy-terminal end [23]. Deletion of exon 2 of *DAX1* was first described by Salvi et al. [23]. The authors reported that deletion of this exon decreases repressor function of DAX1 and also suggest that the deletion of second exon of *DAX1* has most dramatically affected the DAX1 function [23]. Furthermore in mice, deletion of the second exon shows similar symptoms to those observed in humans. On the other hand, *Dax1* knock-out mouse model with deletion of exon 2 revealed testis dysgenesis but normal levels of testosterone and gonadotropins [28].

Immunohistochemical studies of gonadotropins treated testis show moderate to strong immunoreactivity for aromatase in both Leydig and Sertoli cells as well as a strong immunoexpression in Leydig cells for ERα. In addition, high level of estradiol in serum was noted. The DAX1 also represses aromatase production and therefore the production of estrogen [27]. Increased estrogen expression was described in the Leydig cells of *Dax1*-deficient mice [9] and in mice with a partial deletion in the long arm of the Y chromosome [11]. In case of some patients with Sertoli cell-only syndrome increased intratesticular level of estradiol and aromatase expression has been demonstrated [14], as well as strong aromatase expression in Sertoli cells in patient with Klinefelter’s syndrome [12]. However, Brown et al. [4] did not find any evidence for overexpression of aromatase in case of child with AHC. Authors suggest that it may be due to the fact that the protein levels were not high enough to be detected immunohistochemically [4]. It seems that deletion of the second exon of *DAX1* observed in our patient caused absence of its repressor function, and in consequence it leaded to aromatase overexpression and increased estrogen production. Probably, this DAX1 dysfunction through indirect effect is able to disrupt spermatogenesis even with normal testosterone level.

Recent data showed expression of DAX1 in germ cells [15]. Postmortem testicular examination performed at a 23 days-old newborn with AHC and transversion in exon 2 of *DAX1* gene demonstrated a well-defined testis cord containing Sertoli cells, and germ cells surrounded by a interstitial region [4]. These data show that *DAX1* mutation does not disturb normal fetal and neonatal testis development. In the light of this observation, it seems likely that truncated DAX1 protein, due to *DAX1* mutation, expressed in germ cells can direct influence on spermatogenesis failure. Importantly, according to Brown et al. [4] and Frapsauce et al. [5] the process of decay of germ cells increases during time.

Additionally, we showed the presence of FSHR, LHR, ERs, AR in patient testis, but expression of these receptors is not sufficient to induce spermatogenesis by gonadotropins treatment. This lack of spermatogenesis induction is probably associated with disturbed biological function of testis, especially hyperplasia of Leydig cells and a possible defect of Sertoli cells. Interestingly, strong immunostaining of connexin 43 (Cx43) in testis was noted. The importance of Cx43 expression during spermatogenesis has recently been described in humans and several mammalian species [3, 6, 10, 13, 25]. Strong immunostaining, as observed in our case, was detected in the membrane appositions between adjacent Leydig cells in men with Klinefelter’s syndrome [13]. Bravo-Moreno et al. [3] showed that expression of Cx43 in Leydig cells is regulated in an age and functional-dependent manner and suggests that its expression may be participating in the developmental processes required for adequate control of testosterone production and secretion. Normal value of testosterone level after 4 months of gonadotropins treatment in our patient, may indicate an increased metabolic activity of Leydig cells. This observation due to strong signal for Cx43 in Leydig cells may suggests that Cx43 plays important role in the control of Leydig cell function. Furthermore, a strong immunoreactive signal of Cx43 detected between Sertoli cells and remained germ cells may show a role of Cx43 in communication between these cells, as reported previously [3, 25].

To date, few biopsies of testis were performed [4, 5, 20, 21, 24]. Only the biopsy conducted by Frapsauce et al. [5] revealed the presence of few spermatozoa. The first testicular biopsy in patient with AHC and HH due to nucleotide deletion of *DAX1* gene was performed by Seminara et al. *[* 24 *]* after 7 years of low-dose hCG treatment showing few spermatogonia but absence of spermatogenesis. In this patient gonadotropin treatment was sufficient to testicular enlargement, but it was not sufficient enough to induce spermatogenesis [24]. In the case reported by Ozisik et al. [21], testicular biopsy after 6-month treatment in patient with *DAX1* mutation in the N-terminal end demonstrated disorganization of the normal seminiferous tubular structure, and moderate Leydig cell hyperplasia. Another analysis of testicular biopsy assessed by Okuhara et al. [20] after 1 year treatment showed Sertoli cell hypoplasia and no sperm formation.

Up to now, results concerning gonadotropin treatment to the induction of spermatogenesis are unsatisfactory [4, 17, 24]. In the case of our patient, testicular biopsy was performed after 4 months of stimulation and showed lack of spermatozoa. However, Frapsauce et al. [5] was recently reported birth after TESE-ICSI from a man with nonsense mutation in the C-terminal end of *DAX1* and spermatogenesis induced by 20 months of gonadotropins treatment*.* Authors showed that some tubules may contain focal complete spermatogenesis and some spermatozoa may be obtained by testicular biopsy, despite the presence of severe hypospermatogenesis with germ cell arrested at the spermatocyte stage [5]. This report, was emphasized by the authors, gives hope for patients with a mutation in *DAX1* gene after a long treatment with exogenous gonadotropins to obtain children. On the other hand, Mantovani et al. [18] reported that even the year-long gonadotropin treatment did not induce spermatogenesis. However, none of their patients underwent a testicular biopsy, and it is not possible to exclude the presence of a small number of spermatozoa in testis. In order to successful induction of spermatogenesis patients with hypogonadism and mutation in *DAX1* gene should undergo long period of treatment before testicular biopsy [5].

Overall, in the study presented herein we showed overexpression of aromatase in Leydig and Sertoli cells in man with hypogonadotropic hypogonadism associated with adrenal hypoplasia congenita after gonadotropins treatment. The presence of FSHR, LHR, ERs, AR and Cx43 in patient testis suggest that system is functionally efficient and it should be sufficient to induce spermatogenesis. However, our results strongly suggest interrelation between aromatase overexpression leading to increased estrogen production and failure of spermatogenesis induction. In order to confirm and explain our findings more detailed studies need to be undertaken.
